# Personality and Major Depression among Directly Exposed Survivors of the Oklahoma City Bombing

**DOI:** 10.1155/2012/204741

**Published:** 2012-09-13

**Authors:** Carol S. North, C. Robert Cloninger

**Affiliations:** ^1^The VA North Texas Health Care System, Division of Emergency Medicine, Departments of Psychiatry and Surgery, The University of Texas Southwestern Medical Center at Dallas, 6363 Forest Park Road, Suite 651, Dallas, TX 75390-8828, USA; ^2^Department of Psychiatry, Washington University School of Medicine, St. Louis, MO 63110, USA

## Abstract

*Background*. Few disaster studies have specifically examined personality and resilience in association with disaster exposure, posttraumatic stress disorder (PTSD), and major depression. *Methods*. 151 directly-exposed survivors of the Oklahoma City bombing randomly selected from a bombing survivor registry completed PTSD, major depression, and personality assessments using the Diagnostic Interview Schedule for *DSM-IV* and the Temperament and Character Inventory, respectively. *Results*. The most prevalent postdisaster psychiatric disorder was bombing-related PTSD (32%); major depression was second in prevalence (21%). Bombing-related PTSD was associated with the combination of low self-directedness and low cooperativeness and also with high self-transcendence and high harm avoidance in most configurations. Postdisaster major depression was significantly more prevalent among those with (56%) than without (5%) bombing-related PTSD (*P* < .001) and those with (72%) than without (14%) predisaster major depression (*P* < .001). Incident major depression was not associated with the combination of low self-directedness and low cooperativeness. *Conclusions*. Personality features can distinguish resilience to a specific life-threatening stressor from general indicators of well-being. Unlike bombing-related PTSD, major depression was not a robust marker of low resilience. Development and validation of measures of resilience should utilize well-defined diagnoses whenever possible, rather than relying on nonspecific measures of psychological distress.

## 1. Introduction

Most disaster mental health research has focused on PTSD. Less attention has been paid to major depression after disasters, and far less to personality in examining disaster mental health consequences. The role of personality in Axis I psychiatric disorders is well established [1]. Both anxiety and major depressive disorders are associated with high levels of harm avoidance and low self-directedness, but are distinguished by high persistence in those with anxiety and by low persistence in those with depression [1–3].

Another approach to understand the response to stress has been to characterize what distinguishes people who are resilient to stress from those who cope poorly [4]. Resilience is defined as the human ability to adapt and maintain well-being in the face of tragedy, trauma, adversity, hardship, and significant life stressors [5, 6]. High levels of positive and resilient mental health have been attributed to personality traits associated with strong executive functions (such as self-directedness, a self-confident sense of competence and accomplishment, or psychological maturity), social relatedness (such as cooperativeness, social intelligence, social supports), and a positive affective balance (more positive than negative emotions, as with low harm avoidance and high persistence) [7–10]. When studied in samples representative of the general community, such personality traits promote positive well-being and are associated with a lower prevalence of many medical and psychiatric disorders, including anxiety, mood, and stress disorders such as PTSD [11, 12].

Studies of coping and resilience in the general community often accept minor forms of violence and danger as trauma, so that most people are regarded as having experienced posttraumatic stress at some time during their lives [11]. PTSD has sometimes been confused with nonspecific psychological distress characterized by anxiety and depression [13–15]. Anxiety and depression can be nonspecific responses to stress even in people who do not have the avoidance behaviors and numbing symptoms characteristic of PTSD [16]. As a result, it remains unclear whether personality traits or other individual characteristics can differentially predict resilience to PTSD from other forms of psychopathology [17]. 

The 1995 Oklahoma City bombing was the most severe incident of terrorism in the United States at that time. In a study of 182 individuals randomly selected from a registry of directly-exposed survivors of the Oklahoma City bombing studied by this research team, PTSD was found to be the most prevalent diagnosis (34%); major depression was next in prevalence (23%) [16]. Personality in relation to PTSD was also studied in this sample [18] using the Temperament and Character Inventory (TCI) developed by Cloninger's group. Bombing-related PTSD was found to be significantly associated with low self-directedness, high self-transcendence, high harm avoidance, and the combination of low cooperativeness and low self-directedness. The association of PTSD with low cooperativeness, however, fell short of statistical significance (*P* = .065). PTSD was also found to be negatively associated with the creative character configuration and to be positively associated with the disorganized (schizotypal) and autocratic character configurations and the explosive (borderline) temperament configuration. Although the association of preexisting psychiatric illness with bombing-related PTSD was predicted by personality characteristics, the relationship of personality to other disorders such as major depression was not specifically examined [19].

The TCI measures not just maladaptive but also adaptive character and temperament traits [20]. Character reflects the personal goals and values developed by the individual over the lifespan, and temperament represents the emotional core of personality that is largely innate and moderately stable throughout life [20]. Three character dimensions measured by the TCI are self-directedness (i.e., sense of responsibility, purposefulness, and resourcefulness), cooperativeness (i.e., tolerance, helpfulness, and compassion for others), and self-transcendence (i.e., intuitiveness, judiciousness, and spirituality). People with high levels of all three of these character traits have frequent positive emotions and infrequent negative emotions that are, in combination, fundamental to subjective well-being [2]. It is established that the combination of self-directedness and cooperativeness differentiates healthy and disordered personality functioning [20–22].

This paper focuses on personality factors associated with major depression in this sample of Oklahoma City bombing survivors. This study was designed to test the ability of TCI personality dimensions and profiles to differentiate resilience and vulnerability to major depression and PTSD. The causes of both PTSD and major depression are sometimes attributed to stressful life events in general community samples that measure symptomatic distress in a nonspecific way [11, 12]. This study's rigorous measurement of specific diagnostic criteria and multidimensional personality assessment provided the opportunity to determine whether low resilience to postdisaster major depression and PTSD can be differentiated from preexisting psychopathology in a sample with a well-defined, highly traumatic event.

## 2. Methods

The Institutional Review Boards of Washington University School of Medicine and the University of Texas Southwestern Medical Center approved the research. All participants provided written informed consent before participating and were offered $50 in appreciation of their time and effort.

### 2.1. Sample Recruitment and Retention

Survivors who were directly exposed to the Oklahoma City bombing were randomly selected from 1,092 survivors in a bombing survivor registry of the Oklahoma State Department of Health; 71% of those selected agreed to participate in the study, yielding a sample of 182 survivors. This was a highly exposed sample: 87% sustained injuries in the bombing. These survivors participated in research interviews approximately six months after the disaster. Further details on the sampling methods are provided in an earlier publication [16]. The personality assessment was completed by 151 participants (representing 83% of those who completed the research interviews and 59% of those selected from the registry). Completion of the personality measure by study participants was unassociated with gender, age, level of education, marital status, psychiatric diagnoses, injuries, other life events, or treatment received.

### 2.2. Instruments of Assessment

The Diagnostic Interview Schedule/Disaster Supplement (DIS/DS) [23, 24] provided data on psychiatric symptoms and diagnosis, disaster exposure, and other relevant variables including demographic information. The Diagnostic Interview Schedule (DIS) is a fully structured diagnostic interview with acceptable test-retest reliability [25, 26] and interrater reliability in comparisons of DIS with clinician diagnoses [27–29]. “Bombing-related” PTSD refers to PTSD that developed in association with the bombing. Postdisaster prevalence of bombing-related PTSD or major depression is defined as meeting criteria for these disorders at any time since the bombing.

Personality was assessed with the TCI, a self-administered 240-item, true/false self-report instrument measuring three dimensions of character and four dimensions of temperament. More detail about the TCI is available in previous publications demonstrating acceptable interrater reliability and validity in relation to structured interview diagnoses of personality disorder [22, 30]. The TCI character scales are self-directedness, cooperativeness, and self-transcendence. Self-directedness reflects willpower to adapt to or overcome any changes to one's environment. Cooperativeness represents the degree to which a person is agreeable as opposed to self-centered hostility and aggression. Self-transcendence indicates the extent to which one accepts and identifies oneself as an inseparable part of the universe. The TCI temperament scales are harm avoidance, novelty seeking, reward dependence, and persistence. Harm avoidance is the proclivity to avoid signal of punishment, novelty, or frustrative nonreward. Novelty seeking is characterized as attraction to explore novel stimuli, seek excitement, pursue potential rewards, and avoid monotony. Reward dependence indicates pursuit of social attachment based on approval, warmth, and sentimentality. Persistence represents perseverance despite frustration and disappointment.

Character and temperament configuration variables were constructed based on distribution of scale scores either above (high) or below (low) the median across the three character scales and the four temperament scales. For example, disorganized (schizotypal) character type was defined as low in cooperativeness and self-directedness and high in self-transcendence; in contrast, moody (cyclothymic) character type was defined as high in cooperativeness and self-transcendence and low in self-directedness. High levels of cooperativeness and self-directedness are indicators of healthy personal functioning [20, 22]. The occurrence of low cooperativeness and low self-directedness together defines an unhealthy personality structure with underdeveloped executive functions, a core feature of personality disorders [22]. Additional detail about the construction of personality configurations based on TCI scales is available in a previous publication [3].

### 2.3. Data Analysis

SAS Version 9.2 was used for data analysis [31]. Summary results are presented as raw numbers, proportions, means, and standard deviations (SDs). Two dichotomous variables were compared using chi-square tests, and dichotomous variables were compared with numerical variables using Student's *t*-tests. Multiple linear regression models (PROC REG in SAS) were developed to predict dimensional character and temperament dimensions (dependent variables) from other (independent) variables such as psychiatric diagnosis, controlling for sex (because this variable was associated with psychiatric disorders) entered as a covariate independent variable in the models. Multiple logistic regression models (PROC LOGISTIC in SAS) were used to predict dependent dichotomous variables such as psychiatric diagnosis or character and temperament configurations from independent variables including gender entered as a covariate independent variable in the models.

## 3. Results

 The study sample (*N* = 151) was 52% male and 91% Caucasian. The mean age was 43, average education was two and a half years of college, and 66% were married. The most prevalent postdisaster psychiatric disorder was bombing-related PTSD, found in 32%. The next most prevalent disorder was major depression, identified in 21%. Compared to men, women had a higher postdisaster prevalence of both bombing-related PTSD (43% versus 19%, *χ* ^2^ = 9.67, df = 1, *P* = .002) and major depression (33% versus 8%, *χ* ^2^ = 13.2, df = 1, *P* < .001). Controlling for sex as a covariate independent variable entered into a multiple regression model to predict PTSD and major depression (dependent variables) in separate models, neither diagnosis was significantly associated with age, race, marital status, or education also entered simultaneously as independent variables in the model.

Postdisaster major depression was significantly more prevalent among those with (56%) than without (5%) bombing-related PTSD (*χ* ^2^ = 51.79, df = 1, *P* < .001), and 84% of those with postdisaster major depression compared to 5% of those without this disorder also had bombing-related PTSD (*χ* ^2^ = 51.79, df = 1, *P* < .001).

Predisaster major depression was present in 12% of the sample. Postdisaster major depression was significantly more prevalent (*χ* ^2^ = 31.86, df = 1, *P* < .001) among those with (72%) than without (14%) predisaster major depression. Overall, 13% of the sample had an incident major depressive episode (i.e., new cases of major depression after the disaster in people without a predisaster history of the disorder). Most postdisaster major depression (60%) represented incident cases. Significantly more survivors with bombing-related PTSD (33%) than those without (3%) had incident major depression (*χ* ^2^ = 27.55, df = 1, *P* < .001).

Comparisons of character and temperament dimension *t*-scores, controlling for sex, found two scales to be significantly associated with postdisaster major depression: the character dimensions of low self-directedness (mean = 52.6, SD = 9.3 versus mean = 54.9, SD = 6.5; *β* = 5.23, SE = 2.23, *t* = 2.35, *P* = .020) and low cooperativeness (mean = 49.1, SD = 14.7 versus mean = 55.6, SD = 9.5; *β* = 3.01, SE = 1.50, *t* = 2.01, *P* = .046). More of those with postdisaster major depression (50%) than those without (27%) had the combination of low cooperativeness and low self-directedness (controlling for sex differences in a multiple regression model, *β* = 1.13, SE = 0.44; OR = 3.08; 95% CL = 1.30, 7.29; Wald *χ* ^2^ = 6.57, *P* = .010). Similarly, more of those with postdisaster major depression (34%) than those without (13%) had the disorganized (schizotypal) character configuration (*β* = 1.66, SE = 0.53; OR = 5.28; 95% CL = 1.86, 15.00; Wald *χ* ^2^ = 9.74, *P* = .002). Postdisaster major depression was associated with no other character configurations and no temperament configurations. Incident major depression (i.e., postdisaster major depression among individuals without predisaster major depression) was not associated with the combination of low cooperativeness and low self-directedness (present in 32% of those with incident major depression and in 32% of those without; controlling for sex differences, *P* > .05).

A multiple logistic regression model was constructed to predict the combination of low cooperativeness and low self-directedness (dependent variable) from several independent variables entered into the model simultaneously: sex, predisaster and postdisaster major depression, predisaster PTSD, and bombing-related PTSD (see Table 1). In this model, predisaster but not postdisaster major depression and bombing-related PTSD but not predisaster PTSD were independently associated with the combination of low cooperativeness and low self-directedness.

Multiple logistic regression models were constructed to examine the association of postdisaster major depression (dependent variable) with dimensions in character configurations by varying a single dimension (high versus low ranking) while holding the other two dimension rankings constant, controlling for sex differences. Among all the character and temperament configurations examined in these models, only one dimension was associated with postdisaster major depression when holding the other dimensions constant: major depression was associated low cooperativeness when self-directedness was low and self-transcendence was high (*β* = 1.96, SE = 0.86; Wald *χ* ^2^ = 5.21; OR = 7.11; 95% CL = 1.32, 38.30; *P* = .022). Thus, in people with low self-directedness and high self-transcendence, a low level of cooperativeness is the element that associates major depression with the disorganized (scT) character configuration, in contrast to the moody (sCT) character configuration. In a similar logistic regression model substituting incident major depression for postdisaster major depression, this character configuration association with major depression was no longer apparent (*P* > .05). No other dimensions in character configurations and no dimensions in temperament configurations were associated with postdisaster major depression in similar models.

## 4. Discussion

Both bombing-related PTSD and postdisaster major depression were associated with the combination of low cooperativeness and low self-directedness characteristic of unhealthy personal functioning and indicative of personality disorders, particularly the disorganized (schizotypal) character configuration. For major depression, however, these relationships held only for predisaster major depression and were not apparent for incident major depression that developed after the disaster. In contrast, only bombing-related PTSD (not predisaster PTSD related to other traumatic events) was associated with the combination of low cooperativeness and low self-directedness. Thus, personality variables in TCI data, while generally associated with major depression and PTSD in this sample, were specifically associated with development of PTSD related to the bombing but not with development of new cases of major depression after the bombing. Major depression did not reflect the additional associations found between bombing-related PTSD and TCI findings of high self-transcendence, high harm avoidance, and explosive temperament configuration, which seem to be unique to PTSD in these disaster survivors.

In general clinical terms, low resilience to bombing-related PTSD was specifically related to a combination of emotional intensity and instability (i.e., explosive temperament) and magical or superstitious thinking (i.e., high self-transcendence combined with low self-directedness), which results in people having little capacity for emotional self-regulation when stressed. In contrast, people with a downcast character configuration (i.e., low scores in self-transcendence and the other character dimensions) are predisposed to chronic or recurrent depression and anxiety but not PTSD.

Because PTSD is a robust marker of vulnerability to a specific life stressor (trauma), this study's findings strongly indicate that PTSD is particularly useful in validation of the features of psychological resilience in posttrauma situations. Major depression is often associated with PTSD, but its causal determinants are more complex and not specific for assessing resilience in response to specific life stressors. These observations are basic for differentiating the antecedents of well-being in general from those of resilience. Resilience is the ability to adapt and maintain well-being in the face of trauma, adversity, and significant life stressors [5, 6]. Instruments proposed to assess resilience, however, have often been validated using nonspecific measures of psychological well-being or distress with uncertain antecedents [11, 12, 32, 33].

It is well established that PTSD can be distinguished from general psychological distress by the diagnostic requirements of exposure to trauma and the presence of avoidance and numbing symptoms specific to the traumatic event [15, 16, 34–40]. The current analysis has further demonstrated that well-defined PTSD is a robust marker of low resilience. It is doubtful that major depression and the measures of nonspecific psychological distress which are common in the general community provide rigorous indicators of resilience.

Previous work by this research team has shown that rigorously defined PTSD is strongly predicted by specific configurations of temperament and character [18]. Resilience is highest in people with creative character (SCT, i.e., high in self-directedness, cooperativeness, and self-transcendence) who are more resistant to developing PTSD and lowest in disorganized character (scT, i.e., low in self-directedness and cooperativeness, and high in self-transcendence). The presence of high self-transcendence at both extremes of high and low resilience indicates the need to understand the development of well-being and resilience as complex adaptive systems, rather than relying on questionable assumptions about the additive effects of their components [9, 41, 42]. The features that predict a person's positive health may be partly the same and partly different from those that predict resilience to general stress and adversity [6]. PTSD but not major depression should be considered an indicator of resilience to distinguish it clearly from nonspecific measures of mature coping and health.

This study's strengths include the high trauma exposure level of the sample, the random selection of the sample from a disaster registry, and systematic assessment of psychiatric disorders using a structured diagnostic interview in conjunction with TCI data. Findings from this highly-exposed disaster sample may not generalize to survivors of other disasters or to other types of trauma. Despite this study's 17% rate of missing TCI data among study participants, missing TCI data were not associated with any identifiable source of bias in variables related to these analyses. Further details on the potential limitations of this study are provided in a previous publication [18]. It is recognized that associations of TCI scores with postdisaster psychopathology might relate either to effects of preexisting personality characteristics or to changes in personality features following trauma.

## Figures and Tables

**Table 1:** Multiple regression model predicting the combination of low cooperativeness and low self-directedness (dependent variable).

Parameter	df	Estimate	SE	Wald chi-square	*P*	Point estimate	95% Wald CL	
Intercept	1	−1.60	0.36	19.53	<.001			
Male sex	1	0.54	0.40	1.79	.181	1.72	0.78	3.78
Predisaster major depression	1	1.23	0.60	4.16	.041	3.42	1.05	11.11
Postdisaster major depression	1	1.00	0.58	0.00	1.00	1.00	0.32	3.12
Predisaster PTSD	1	0.10	0.53	0.04	.843	1.11	0.40	3.12
Bombing-related PTSD	1	1.14	0.48	5.78	.016	3.13	1.24	7.95
